# Draft genome sequences of *Weissella cibaria* GM93m3, a promising probiotic strain from raw goat milk

**DOI:** 10.1128/mra.00270-24

**Published:** 2024-07-03

**Authors:** Muiz O. Akinyemi, Oluwawapelumi A. Oyedele, Mariska S. Kleyn, Bukola A. Onarinde, Rasheed A. Adeleke, Chibundu N. Ezekiel

**Affiliations:** 1 National Centre for Food Manufacturing, University of Lincoln, Lincoln, United Kingdom; 2 Unit for Environmental Sciences and Management, North-West University (Potchefstroom Campus), Potchefstroom, South Africa; 3 Department of Microbiology, Babcock University, Ilishan Remo, Ogun State, Nigeria; 4 Department of Agrobiotechnology (IFA-Tulln), Institute of Bioanalytics and Agro-Metabolomics, BOKU University, Vienna, Austria; Department of Biological Sciences, Wellesley College, Wellesley, Massachusetts, USA

**Keywords:** probiotics, *Weissella*, gut health, food safety

## Abstract

The draft genome of a previously documented potential probiotic *Weissella cibaria* strain GM93m3 from raw goat milk in Nigeria is reported. The total genome size was 2,447,229 with 46 contigs and G+C content of 44.86%.

## ANNOUNCEMENT

*Weissella cibaria,* a Gram-positive, rod-shaped, non-motile, lactic acid bacterium, has attracted research interest due to its technological, nutritional, and probiotic potential (1). *W. cibaria* GM93m3, isolated from raw goat milk collected in June 2019 from dairy farms in Sokoto state, Nigeria (13.0533°N, 5.3223°E), was proposed as a suitable human probiotic candidate in a prior study. The strain showed *in vitro* the ability to tolerate human gastrointestinal conditions and co-aggregate with pathogens and antioxidant activities (1). Here, we report the draft genome sequence of this *W. cibaria* GM93m3 strain.

Milk samples were serially diluted in threefolds, inoculated on De Man–Rogosa–Sharpe agar, and incubated anaerobically at 37°C for 24 hours. Distinct colonies were subcultured until pure culture was attained.

High-quality genomic DNA was extracted from pure single colonies of the strain using the Quick-DNA fungal/bacterial miniprep kit (Zymo Research). A genomic library was prepared using the Illumina TruSeq Nano DNA library preparation kit supplied by Illumina Inc. Pair-end sequencing (2 × 150 base pairs) was performed using Illumina NovaSeq 6000 generating 9,224,104 total read counts. The quality of sequence reads was determined using FastQC v0.12.0 (2). Adapter trimming, quality filtering, and per-read quality pruning were done using fastp v0.23.4 (3). Filtered paired-end reads were *de novo*-assembled using SPAdes v3.15.3 (4). Quality of assemblies was determined using QUAST v4.4 (5), while genome completeness was evaluated using CheckM v1.0.18 (6).

**Fig 1:**
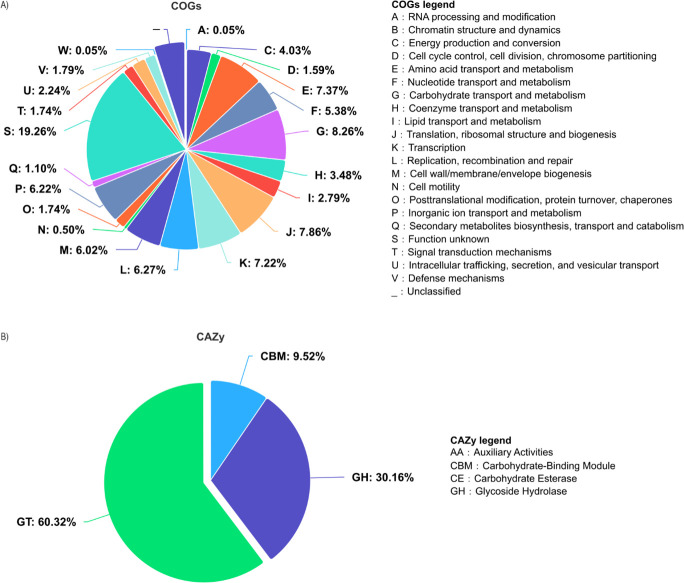
(**A**) COGs functional classification and (**B**) CAZy annotated for *Weissella cibaria* GM93m3 strain.

GM93m3 sequence (PRJNA1086807) was assembled into 46 contigs with a genome size of 2,447,229 bp, N50 of 235,178, L50 of 4, G+C content of 44.86%, and coverage of 570.09. CheckM analyses indicated that the genome was 100% complete with no contamination. Species designation of the strain was first determined using 16S rRNA gene extracted from the genome using extractseq version 5.0.0 (7) and blasted against the National Center for Biotechnology Information 16S database (8). The closest hit, which corresponds to *W. cibaria* strain SRCM103448 (CP035267.1), exhibited an identity similarity of approximately 98%. Further analysis identifying closely similar reference genomes and orthologous average nucleotide identity (ANI) calculated using the Mash/MinHash v2.3 algorithm (9) revealed 98.37% ANI with *W. cibaria* KACC 11862 (GCA_000193635.2). Functional genome annotation by orthology was performed using eggnog-mapper software v2.1.8 and database v5.0.2 with protein coding sequences (CDSs), translated to proteins before the search identified a total of 2,257 CDSs, 74 transfer RNA genes, 8 ribosomal RNA, and 1 transfer-messenger RNA genes. An overview of clusters of orthologous genes (COGs) functional classification and carbohydrate-active enzymes (CAZy) is presented in Fig. 1 while the genome annotation and 16S rRNA gene are available on Figshare (10). ABRicate v1.0.1 (11) was used to examine plasmids, virulence determinants, and resistance-related genes, with reads aligned to the PlasmidFinder (12), Virulence Factors (13), and Comprehensive Antibiotic Resistance v3.2.9 (14) databases, respectively; however, no hits were detected. The BAGEL v.4.0 webserver (http://bagel4.molgenrug.nl) predicted the absence of genes encoding bacteriocins, which agrees with our previous phenotypic report where the supernatants of strain GM93m3 showed the least antimicrobial activity against selected foodborne pathogens (1). Default parameters were used for all software unless otherwise specified.

## Data Availability

This Whole Genome Shotgun project has been deposited at DDBJ/ENA/GenBank under the accession JBBEEN000000000. The version described in this paper is version JBBEEN010000000. The raw data of our study are available in the SRA database of NCBI with accession number SRX23974901.
